# Parliament and the Profession

**Published:** 1916-03-11

**Authors:** 


### PARLIAMENT AND THE PROFESSION.

### The Advisory Medical Board.

The unofficial members of the Advisory Board for Army Medical Services are Professor Kenwood, Sir John Rose Bradford, Professor Leonard Hill, Sir Anthony Bowlby, and Sir Charles Cameron. A statement to this effect was made by Mr. Tennant in reply to Mr. Lynch, who asked whether Sir A. Bowlby and Sir J. R. Bradford sat on the board as civilian representatives while holding military posts at the time. Mr. Tennant added that they were not put on the board as representing "civilian interests."

### Territorial Base Hospitals.

Civilian medical officers are employed to a very small extent in Territorial Force general hospitals, as these institutions are staffed by officers of the Territorial Force. The number of Territorial Force officers mobilised varies with the number of patients in the wards, and Mr. Tennant stated in the House of Commons that in the base hospitals in France the number of medical officers now employed had, in the view of the military authorities, reached an irreducible minimum.

### Hospital Accommodation for Wounded Soldiers.

Some interesting figures with regard to the hospital accommodation in London for wounded soldiers were given by Mr. Tennant in reply to a question by Mr. Percy Harris. On February 12 last there were in military hospitals in London 2,437 wounded soldiers, in auxiliary hospitals 1,267, and in civil hospitals 578. In the military hospitals on the same date there were 5,346 vacant beds, a fact which no doubt accounts for the decision of the authorities to transfer some of the wounded soldiers from voluntary to military hospitals.

### R.A.M.C. Prisoners in Germany.

Answering a question by Mr. Hewins, Lord Robert Cecil stated that Major Priestly and Captain Vidal, of the Royal Army Medical Corps, are still detained in Germany in violation of the provisions of the Geneva Convention. He could only explain the action of the German Government by the supposition that the treatment of these two officers had been such, and the state of affairs in the Wittenberg Camp, particularly during the typhus epidemic, so bad, that the German Government considered it undesirable to afford them the opportunity of reporting on these matters to the War Office. (These officers have since been released.)

### The Distribution of Medical Officers at the Front.

It was suggested by Mr. Lynch that on a recent occasion it happened that some medical officers were employed at the Front for fifty-two hours at a stretch, while the medical officers of another division were completely unemployed, but the Under-Secretary for War said he had no knowledge of that fact. It was, he added, the duty of the higher military authorities to distribute the medical officers under their command so as to meet the various exigencies which might arise. He agreed, however, that such a contingency might happen as the one mentioned by Sir A. Markham, who alleged that when a certain cavalry corps reached the trenches there was only one medical officer to attend to all casualties.

### Morphine for the Army.

Opium is of special importance for the treatment of the wounded, and in view of the great demand for this drug and of the special conditions affecting the usual sources of supply at the present time, it is not considered that the present and prospective stocks are in excess of what may reasonably be regarded as necessary for the requirements of the country. This statement was made by Mr. Bridgeman, as representing the War Trade Department, in reply to Mr. Samuel Samuel, who suggested that the re-exportation of opium was prohibited in order to accumulate stocks of unsold opium with a view to manufacturers of morphine buying it at their own prices later on. As the price of morphine is regulated by the cost of opium, it is difficult to see what advantage the manufacturers would enjoy if, as a result of the accumulation of stocks, the price of opium was substantially reduced.

### Other Matters of Interest.

The Recording of Military Cases.—Replying to Mr. Thorne, Mr. Tennant stated that the recording of cases was part of the established routine of every military hospital.

Movable Hospitals at the Front.—Replying to Mr. Lynch, Mr. Tennant stated that movable hospitals had been in use at the Front for some time.

Blind Soldiers.—The number of soldiers who have been rendered totally blind in the present war is larger than that stated by Mr. Tennant a week ago (The Hospital, March 4, p. 512). Since then Mr. C. A. Pearson, the chairman of the Blinded Soldiers and Sailors' Care Committee, has supplied figures showing that the total number of non-commissioned officers and men discharged from the Army for blindness is 156. There are also nine British officers who have lost their sight at the Front.

New Zealand Military Hospitals.—Sir A. Markham drew attention to a statement made in the New Zealand Parliament by the Minister of Defence to the effect that cables to the Army Council offering to provide hospitals for the New Zealand Expeditionary Force in Gallipoli had not been answered, and that a cable addressed to Lord Kitchener had only been replied to after a long delay. Mr. Asquith explained that the delay in replying was due to the fact that it had been necessary in the interval to consult the military authorities in Egypt before any proposals for bringing additional *personnel* were confirmed.

